# Diagnosis of spontaneous bacterial peritonitis in cirrhotic patients in northeastern Brazil by use of rapid urine-screening test

**DOI:** 10.1590/S1516-31802006000300006

**Published:** 2006-05-04

**Authors:** Lucia Libanez Bessa Campelo Braga, Marcellus Henrique Loiola Ponte de Souza, Alzira Maria de Castro Barbosa, Felipe Magalhães Furtado, Paula Andréa Maia Campelo, Antônio Haroldo de Araújo

**Keywords:** Cirrhosis, Ascites, Diagnosis, Peritonitis, Reagent strips, Urinalysis, Cirrose, Ascite, Diagnóstico, Peritonite, Fitas reagente, Urinálise

## Abstract

**CONTEXT AND OBJECTIVE:**

Spontaneous bacterial peritonitis (SBP) is a frequent and severe complication of cirrhotic patients with ascites. It has been proposed that the reagent strip for leukocyte esterase designed for the testing of urine (Combur test^®^ UX) could be a useful tool for diagnosing SPB. The aim of this study was to assess the sensitivity and specificity of urine test strips for diagnosing SBP in cirrhotic patients with ascites.

**DESIGN AND SETTING:**

Prospective study, at a university hospital in northeastern Brazil.

**METHODS:**

Forty-two unselected consecutive cirrhotic patients (32 males; mean age: 51.7 ± years) were included, and a total of 100 paracenteses were performed. All ascitic fluid samples were analyzed using the reagent strip and cytology, neutrophils, lymphocyte count, appropriate biochemical tests and culturing. The strips were considered positive if the color became purple on a colorimetric scale.

**RESULTS:**

Nine patients were diagnosed with SBP using cytology (> 250 neutrophils/mm^3^), and the strips were positive for all these nine patients with SBP. In one sample, the strip was positive but the neutrophil count was less than 250 cells/mm^3^. For 86 samples, both the strips and cytology were negative. At the threshold of 250 neutrophils/mm^3^ in ascitic fluid, the sensitivity, specificity, positive predictive value and negative predictive value for the strips were respectively 100%, 98.9%, 92.3% and 100%.

**CONCLUSION:**

The Combur test^®^ UX urine screening test is a very sensitive and specific method for diagnosing SBP in cirrhotic patients with ascites.

## RESUMO

**CONTEXTO E OBJETIVO:**

Peritonite bacteriana espontânea (PBE) é uma freqüente e grave complicação em pacientes cirróticos com ascite. Vem sendo proposto o uso de fitas reagente para esterase leucocitária, utilizadas para diagnóstico de infecção urinária, como instrumento para diagnosticar PBE. O objetivo deste estudo é avaliar a sensibilidade e a especificidade da fita reagente Combur Test^®^ UX no diagnóstico de PBE em pacientes cirróticos com ascite.

**TIPO DE ESTUDO E LOCAL:**

Estudo prospectivo, realizado no Hospital Universitário Walter Cantídio, Fortaleza, Ceará, Brasil.

**MÉTODOS:**

Quarenta e dois pacientes consecutivos, não selecionados (32 homens, com média de idade de 51,7 ± anos) foram incluídos e um total de 100 paracenteses foram realizadas. O líquido ascítico foi avaliado pela fita reagente (Combur Test^®^ UX), citologia, contagem de neutrófilos, linfócitos, testes bioquímicos e cultura. As fitas reagentes foram consideradas positivas se havia mudança na cor para roxo na escala colorimétrica fornecida pelo fabricante.

**RESULTADOS:**

Foram diagnosticados nove pacientes com PBE pela citologia (> 250 neutrófilos/mm^3^), estando a fita Combur Test^®^ UX positiva em todos os nove pacientes. Em uma amostra o teste da fita foi positivo, embora a contagem de neutrófilos fosse < 250 células/mm^3^. Nas outras 86 amostras ambos os testes foram negativos. Tendo-se como ponto de corte 250 PMN/mm^3^ no líquido ascítico, a sensibilidade, a especificidade, o valor preditivo positivo e o valor preditivo negativo foram respectivamente 100%, 98,9%, 92,3% e 100%.

**CONCLUSÕES:**

Combur Test^®^ UX (fita reagente para urinanálise) é um método sensível e específico para o diagnóstico de PBE em pacientes cirróticos com ascite.

## INTRODUCTION

Spontaneous bacterial peritonitis (SBP) is a frequent and severe complication of cirrhotic patients with ascites. The prevalence of SBP among unselected hospitalized cirrhotic patients with ascites ranges between 10% and 30%.^1–5^ Although antibiotic therapy produces a good response, the mortality rate due to SBP remains high: approximately 30%-50%.^6–9^ Improved survival in SBP episodes might be obtained through rapid diagnosis and treatment.

The polymorphonuclear (PMN) leukocyte count in the ascitic fluid has proven to be a very useful and sensitive method for diagnosing SBP.^10–15^ SBP is highly likely when the PMN cell count in the ascitic fluid reaches a cutoff of 250/mm^3^.^10^ Once this cut-off has been reached, antibiotic therapy must be started immediately, without waiting for a culture from the ascitic fluid. However, total and PMN leukocyte counts from the ascitic fluid are not always available everywhere, or it may be impossible to obtain them in emergency situations.

Reagent strip testing for leukocyte esterase has been found to be a sensitive and accurate predictor for the presence of PMN in body fluids such as urine,^16–18^ cerebrospinal fluid, seminal and peritoneal fluid.^19^ This test is based on the esterase activity of granulocytes present in the biological fluid, which reacts with a chemical compound on the reagent strip to cause a color change in the azo dye (purple).

It has been proposed that reagent strip testing for leukocyte esterase could be utilized to reduce the time between performing paracentesis and obtaining a presumptive diagnosis of SBP from a few hours to a few seconds. Moreover, such strips would be available everywhere, and could be a useful tool for diagnosing SBP, especially in developing countries. Therefore, it seems very important to validate the use of such reagent strips in a country like Brazil, and especially in the northeast of this country.

## OBJECTIVE

The aim of this prospective study was to evaluate the accuracy of reagent strips for rapidly diagnosing SBP in cirrhotic patients with ascites in the university hospital in Fortaleza, State of Ceará, Brazil.

## PATIENTS AND METHODS

### Patients

This study was carried out in Hospital Universitário Walter Cantídio between March and August 2004 and it was approved by the Ethics Committee of Hospital Universitário Walter Cantídio, Universidade Federal do Ceará. Forty-two consecutive unselected cirrhotic patients (32 males, 10 females, mean age 51.7 years) were included and a total of 100 paracenteses were performed. The diagnosis of cirrhosis was established by histological criteria or by clinical criteria (splenomegaly, ascites and/or esophageal varices) and ultrasonography findings. All these patients had evidence of advanced liver disease: 23 patients (54.7%) were in Child-Pugh class B and 19 patients (45.3%) in Child-Pugh class C. The cirrhosis was related to alcoholism in 27 patients (64.2%), and to chronic hepatitis B or C in six patients (14.2%). The characteristics of the study population are summarized in Table 1.

**Table 1.** Characteristics of 42 patients with cirrhosis who underwent 100 paracenteses

Characteristics	Mean ± SD or number (%)
Female sex	10 (23.8%)
Age (mean ± SD)	51.7
Child-Pugh classification	
A	0 (0%)
B	23 (54.8%)
C	19 (45.2%)
Etiology	
Viral hepatitis	6 (14.3%)
Alcohol abuse	27 (64.3%)
Alcohol + viral hepatitis	1 (2.4%)
Others	8 (19.0%)

*SD = standard deviation.*

The diagnosis of SBP was defined as a PMN count in the ascitic fluid greater than 250/mm^3^, in the absence of a contiguous source of intra-abdominal infection, with or without a positive culture,^10^ and after excluding other causes of elevated PMN in ascitic fluid such as tuberculosis, peritoneal carcinomatosis or pancreatitis. Antibiotic therapy was started empirically in all cirrhotic patients with an ascitic fluid PMN cell count greater than 250/mm^3^ and consisted of 5-10 days of ceftriaxone (2 g/day).^10^ One follow-up paracentesis was performed after two days of antibiotic therapy to determine the PMN count in the ascitic fluid.

### Paracentesis

Paracentesis was performed under aseptic conditions in the cirrhotic patients at the time of admission to hospital, or routinely in an outpatient setting, to investigate ascites. Three samples of ascitic fluid were obtained from each patient. Ascitic fluid was classically processed including investigation of cytology, PMN leukocytes, lymphocyte count and appropriate biochemical tests. The ascitic samples for PMN leukocytes and total leukocyte count were collected in tubes containing ethylenediaminetetraacetic acid (EDTA) anticoagulant. Cytological examinations and differential cell counts were performed using a conventional optical microscope.

Culturing of ascitic fluid was performed by inoculating 5 ml of ascitic fluid into 3 ml of BHI (brain heart infusion), and into MacConkey agar and chocolate agar.

### Combur Test^®^

Immediately after the paracentesis, the ascitic fluid was tested by using a reagent strip for leukocyte esterase designed for the testing of urine (Combur Test^®^ UX; Roche Diagnostics GmbH, D-68298 Mannheim, Germany). The procedure utilized was the same as described by the manufacturer for urine. Briefly, the sample of fresh ascitic fluid was collected in a clean, dry container. All reagent areas were immersed in the ascitic fluid and the strip was removed immediately. The strip was read by the physician who carried out the paracentesis (who was unaware of the results from the cytological and bacteriological tests). The color of the leukocyte reagent area was then compared with the color chart on the bottle, and the result was scored on a colorimetric four-grade scale (0-3). The correlation between PMN and the four-grade scale suggested by the manufacturer was as follows: grade 0: 0 PMN/mm^3^; grade 1: 25 PMN/mm^3^; grade 2: 75 PMN/ mm^3^; grade 3: 500 PMN/mm^3^. The reagent strip was considered positive if the color turned to purple, i.e. if the grade was 2 or 3. We considered the strip positive when it showed grade 2 or 3 on the basis of previous studies that demonstrated that the sensitivity of the test improved with this cutoff.^20,21^ Since SPB is an infectious disease with high mortality, it is important to have a test with high sensitivity.

### Statistical analysis

Quantitative variable are expressed as the mean ± standard deviation (SD). The sensitivity, specificity, positive predictive value and negative predictive value were determined.

## RESULTS

One hundred paracenteses were performed on 42 cirrhotic patients from March 2004 to August 2004 in our primary referral hospital.

Sixty-five paracenteses were performed routinely in an outpatient setting to investigate refractory ascites, and 35 during classical hospitalization. Paracenteses were systematically performed on 27 asymptomatic inpatients to investigate new onset of ascites and to confirm symptoms suggestive of peritoneal infection in the other eight inpatients. The number of paracenteses per patient ranged from one to eight, with an average of two.

During the study period, SPB was diagnosed in nine patients by cytology (> 250 neutrophils/mm^3^); however, only three of these nine ascitic fluid cultures grew. The test with the strips (Combur Test^®^ UX) was positive (colorimetric scale 2 or 3) for all nine patients with SBP (Table 2). The paracentesis was repeated after 48 hours on antibiotics, for four patients with SBP. In three cases, the neutrophil count remained above 250/mm^3^ and the strips were also positive: in these cases, the antibiotics were changed. In the other case, the neutrophil count was below 250/mm^3^ and the strip was negative. In five patients with SBP, it was not possible to collect ascitic fluid after 48 hours on antibiotics, because one patient died, two patients no longer had any ascitic fluid, and two patients abandoned the treatment.

**Table 2.** Details regarding of spontaneous bacterial peritonitis detected by cytology and strip test in nine cirrhotic patients

Patient	In or outpatient	Strip result	Polymorphonuclear cell count (cell/mm^3^)	Ascitic fluid culture result
1	Inpatient	2	300	0
2	Inpatient	2	504	0
3	Inpatient	3	28560	*Escherichia coli*
4	Inpatient	2	553	0
5	Inpatient	3	5133	*Pseudomonas aeruginosa*
6	Inpatient	2	260	0
7	Inpatient	3	5004	*Escherichia coli*
8	Outpatient	2	2592	0
9	Inpatient	2	1675	0

In one sample, the test with the strip was positive but the PMN count was below 250/mm^3^. In this case, the ascitic fluid culture was also negative. In the other 86 samples of ascitic fluid, the neutrophil counts were below 250/mm^3^ and the strip tests were negative in all cases.

At the threshold of 250 PMN/mm^3^ in ascitic fluid, the sensitivity, specificity, positive predictive value and negative predictive value were respectively 100%, 98.9%, 92.3% 100%, as shown in Table 3.

**Table 3.** Sensitivity, specificity, positive and negative predictive value for the diagnosis of spontaneous bacterial peritonitis in 42 cirrhotic patients (100 paracenteses) by using the Combur Test^®^ UX reagent strip

Variable	Number	Value
Sensitivity	12/12	100%
Specificity	87/88	98.9%
Positive predictive value	12/13	92.3%
Negative predictive value	87/87	100%

## DISCUSSION

This prospective study confirmed the high accuracy of reagent strips for the diagnosing of SBP in cirrhotic patients with ascites. Our results showed that a grade 2 or 3 result from the Combur test^®^ UX reagent strip had highly positive predictive value (92.3%), with a specificity of 98% for the SBP diagnosis. On the other hand, in patients with a reagent strip result of grade 0 or 1, a diagnosis of ascitic fluid infection could confidently be ruled out because the negative predictive value was 100%. Our results are consistent with previous reports. Vanbiervliet et al. showed that the Multistix 8SG rapid urine screening test had 100% sensitivity and specificity for SBP diagnosis.^22^ More recently, Castellote et al. demonstrated that another urine screening test (Aution sticks) also had high sensitivity (96%) and specificity (89%) for detecting SBP in cirrhotic patients with ascites.^20^ In another study, Théovenot et al. tested the reagent Combur-2 test^®^ LN, and showed a sensitivity of 89% and a specificity of 100%.^21^

In the present study, one false positive result was found. Despite the fact that a false positive result could indicate antibiotic therapy for a patient without SBP, this therapy would be discontinued if the PMN count were below 250 cell/mm^3^. Considering the high mortality from SBP episodes, a method with high sensitivity is essential for the management of these patients.

The reagent strips are easy to use, do not require expertise, are rapid, can be performed everywhere, and have high sensitivity and specificity for diagnosing of SBP, thereby allowing antibiotic therapy to immediately start. Another noteworthy point is the low cost of these strips (R$ 0.15 per strip). This new diagnostic method is also useful for determining the effectiveness of the antibiotic therapy. In fact, there were three SBP episodes in which we observed that the PMN count remained above 250/mm^3^ after 48 hours of ceftriaxone therapy, and these positive results were always confirmed by the PMN cell count.

The incidence of SBP in our study was 10.4%, which was lower than previously reported.^10^ This difference can be explained by the greater number of outpatients in our study (82%).

In the present study, only three positive cultures were observed among the nine cases of SBP (33%). It is possible that this low rate was due to suboptimal culturing methods and techniques. However, like what was found in this study, low rates of positive SBP cultures have been reported in other studies, with proportion ranging from 39% to 59%,^12,23-25^ and these findings probably reflect earlier diagnosis of the infection. All three patients for whom the cultures were positive were inpatients and had advanced liver disease, and in one of them *Pseudomonas aeruginosa* was isolated, a germ that is resistant to ceftriaxone. The source of the SBP seems to have to the therapy planning, since nosocomial organisms are usually more resistant to antibiotics than those acquired in the community. Therefore, it is worthwhile culturing ascitic fluid in inpatients with advanced diseases.

The simplicity and low cost of this test is an important point in our results. Many hospitals in our country have limited laboratory facilities, or are unable to perform PMN counts in ascitic fluid at night or over the weekend. Considering the mortality from SBP, this test will help to improve the management of SBP.

## CONCLUSIONS

In summary, this is the first study using this low-cost method for diagnosing SBP in northeastern Brazil. Our results are comparable with those reported previously, thus demonstrating that this accurate method could be used everywhere, thereby reducing the time from paracentesis to a presumptive diagnosis of SBP from a few hours to a few seconds.
